# Feeding rumen-protected choline during the periconceptional period programs postnatal phenotype of suckled beef calves

**DOI:** 10.1186/s40104-025-01188-8

**Published:** 2025-04-02

**Authors:** Masroor Sagheer, McKenzie L. J. Haimon, Samuel Hincapie Montoya, Daniella Heredia, Federico Tarnonsky, Mauro E. Venturini, Angella Gonella-Diaza, Nicolas DiLorenzo, Joseph W. McFadden, Gabriela Dalmaso de Melo, Ky G. Pohler, Peter J. Hansen

**Affiliations:** 1 Department of Animal Sciences, D.H. Barron Reproductive and Perinatal Biology Research Program, and Genetics Institute, University of Florida, Gainesville, FL USA; 2 Present Address: Division of Reproductive Endocrinology & Fertility, Department of Obstetrics & Gynecology, University of Florida, Gainesville, FL USA; 3 Present Address: Department of Animal Science, University of Wyoming, Laramie, WY USA; 4 North Florida Research and Education Center, University of Florida, Marianna, FL USA; 5 Department of Animal Science, Cornell University, Ithaca, NY USA; 6 Department of Animal Science, Texas A&M University, College Station, TX USA

**Keywords:** Beef cattle, Choline, Pregnancy, Programming

## Abstract

**Background:**

Supplementation of choline chloride in culture medium programs the preimplantation bovine embryo to increase weaning weight of the resultant calf. Here, it was hypothesized that similar programming actions of choline can be induced by feeding rumen-protected choline (RPC) to beef cows during the periconceptional period.

**Results:**

A preliminary experiment was conducted to determine changes in circulating concentrations of choline and its metabolites after RPC supplementation. Suckled beef cows were individually fed 0, 30, 60, and 90 g of RPC (i.e., 0, 8.6, 17.3 and 25.9 g choline chloride) and blood samples were collected at random times after feeding. There were no differences in plasma concentrations of choline or its metabolites between groups. In the second experiment, effects of feeding 60 g/d RPC from d −1 to 7 relative to timed artificial insemination were examined for suckled beef cows. Feeding RPC did not affect pregnancy or calving rates, pregnancy losses, plasma concentrations of pregnancy-associated glycoproteins, gestation length or calf birth weight. Calves from RPC fed dams were lighter than control calves at ~118 days of age (range 75–150; age included in the statistical model) and at weaning at ~248 days of age. There was no effect of treatment on hip height at ~118 days of age although there was a trend for RPC calves to be shorter at weaning. Weight/height ratio was lower for RPC than control at both 118 and 248 days of age. Treatment did not affect testis weight at ~118 days of age.

**Conclusions:**

Supplementation of RPC during the periconceptional period programmed development to alter calf phenotype in the postnatal period. The net result, reduced body weight, was the opposite of the phenotype caused by the addition of choline to embryo culture medium.

## Background

Developmental programming is the phenomenon whereby changes in the environment of a developing organism, or of the gametes from which it is derived, causes changes in development to affect postnatal phenotype. Although the exact mechanism of developmental programming is unknown, epigenetic programming involving DNA methylation and histone modifications has been suggested to play an important role in altering postnatal phenotype [1]. Developmental programming can occur in the periconceptional period of development as indicated by programming actions of a low-protein diet [2], lipopolysaccharide exposure [3], and actions of embryokines such as colony-stimulating factor 2 [4], and dickkopf WNT signaling inhibitor 1 [5].

Choline is another molecule that can program embryonic development, at least in the cow. Choline is a water-soluble, vitamin-like nutrient that is transported into the cell through solute carrier 44A [6] where it can be metabolized by several different pathways including acetylation in the presence of acetyl-CoA by choline acetyltransferase to form the neurotransmitter acetylcholine, phosphorylation by choline kinase for generation of phosphatidylcholine and sphingomyelin through the Kennedy pathway, and oxidization by choline oxidase to produce betaine for donation of methyl groups for DNA methylation through generation of *S*-adenosylmethionine [7]. There are also reports that choline can regulate the AMPK and mTOR cell-signaling pathways in a context-specific manner [8, 9]. Choline has been demonstrated experimentally to function as a methyl donor in the cow [10].

Evidence that choline can program development during the preimplantation period comes from studies with bovine embryos from Brahman and Brangus donors produced in vitro [11–13]. When cultured in serum-free conditions, such embryos are usually cultured in a medium without choline. Addition of choline chloride to culture medium, at a final concentration of either 4 µmol/L [11] or 1.8 mmol/L [12, 13] changed the developmental program of the embryo so that the resultant calves experienced greater postnatal growth, higher weaning weight and specific changes in DNA methylation in blood and *longissimus thoracis* muscle.

Discovery that choline can act on the preimplantation embryo to have beneficial effects on phenotype of the postnatal animal leads to the prospects of feeding choline to female animals during the period of early pregnancy to enhance productivity of the offspring. In ruminant species, bacterial trimethylamine lyases degrade choline in the gut to generate trimethylamine which is converted into trimethylamine *N*-oxide (TMAO) in the liver by flavin-containing monooxygenase 3 [14]. Several rumen-protected choline (RPC) products have been developed which utilize fat coatings to partially protect choline from degradation by gut microbes.

The objective of the current study was to evaluate whether feeding RPC to cows during the periconceptional period can alter the postnatal phenotype of calves. It was hypothesized that feeding RPC to beef cows from d −1 to 7 relative to timed artificial insemination (TAI) programs the embryo in a manner that increases postnatal growth of the calves. One initial experiment was performed to determine changes in plasma concentrations of choline and its metabolites after feeding RPC. Subsequently, it was tested whether individual feeding of RPC from d −1 to 7 relative to TAI programs postnatal development.

## Methods

### Animal care

All procedures involving animals were approved by the Animal Care and Use Committee of the University of Florida and were performed following the relevant guidelines and regulations.

### Circulating concentrations of choline and its metabolites in cows fed in a self-feeding device (Exp. 1)

The experiment was designed to quantify the plasma concentrations of free choline and its metabolites after feeding different levels of RPC [ReaShure, Balchem Inc., New Hampton, NY, USA; 28.8% choline chloride (w/w)]. A total of 24 postpartum suckled beef cows housed in a single pasture were randomly assigned to be individually fed either 0 (*n* = 5), 30 (*n* = 7), 60 (*n* = 7), or 90 (*n* = 5) g of RPC (i.e., 0, 8.6, 17.3 and 25.9 g choline chloride). The RPC was mixed in 454 g of ground corn gluten. Treatment diets were fed individually for 9 d using a single Super SmartFeed machine (C-Lock, Rapid City, SD, USA). Before the provision of the experimental diet, all cows were fed the control diet (ground corn gluten) using Super SmartFeed for ~21 d for training purposes. The machine features four stainless-steel feed bins, a radio frequency identification (RFID) reader, an antenna, an adjustable metal framework to limit access to one animal at a time, and a data acquisition system that records RFID tags and feed bin weight. Each bin contained one specific diet corresponding to 0, 30, 60 or 90 g of RPC. The feed would only be released to a cow if it approached a feeding bin for the assigned diet. For each visit to the bin, the system recorded the cow RFID, bin number, initial and final times, and beginning and ending mass in the bin to allow calculation of the exact amount of feed intake by each cow. The machine was programmed to allow each cow to consume no more than 454 (0 g RPC), 484 (30 g RPC), 514 (60 g RPC), and 544 g (90 g RPC) of the diet each day. The daily feed intake recorded by the Super SmartFeed machine averaged 386 g for 0 g RPC (quartiles 1, 2 and 3 = 386, 437, and 437 g), 331 g for 30 g RPC (quartiles 1, 2 and 3 = 132, 484, and 484 g), 396 g for 60 g RPC (quartiles 1, 2 and 3 = 392, 490, and 490 g) and 447 g for 90 g RPC (quartiles 1, 2 and 3 = 484, 521, and 521 g).

Blood samples (10 mL) were collected by jugular venipuncture at d −1, 5, 5.5, 10, and 10.5 of feeding into EDTA-containing tubes and immediately centrifuged at 3,000 × *g* for 15 min to obtain the blood plasma fraction. Note that cows could eat ad libitum until the daily allocation of feed had been consumed so timing of the collection of blood was not standardized to feed consumption. Plasma samples were stored at −80 °C until further analysis. Concentrations of choline, betaine, dimethylglycine, methionine, and TMAO in plasma were determined by liquid chromatography coupled with tandem mass spectrometry [15] while plasma concentrations of phosphatidylcholine, sphingomyelin, and lysophosphatidylcholine were measured by liquid chromatography-mass spectrometry [16].

### Programming of postnatal development by feeding of rumen-protected choline in the periconceptional period (Exp. 2)

The experiment was designed to test whether feeding RPC [ReaShure; 28.8% choline chloride (w/w)] during the periconceptional period can affect pregnancy outcomes and alter the postnatal development of the resultant calves. Due to the limitation in the number of cows that can be assigned to one Super SmartFeed machine, the experiment was performed in three replicates. A total of 328 postpartum, first-service suckled Angus and Brangus cows were available for the experiment (*n* = 117, 124 and 87 for replicates 1, 2 and 3). Before the provision of the experimental diet for each replicate, cows were placed in a single pasture and given access to 454 g/d of ground corn gluten using a single Super SmartFeed for ~21 d for training purposes. Only cows that consistently consumed the diet from the Super SmartFeed (i.e., consumed ~454 g of the diet) were retained for the experimental period. Accordingly, 218 postpartum first-service suckled Angus and Brangus cows entered the experiment (*n* = 67, 82, and 69 for replicates 1, 2 and 3). For each replicate, cows within each breed were blocked by body weight and assigned randomly within the block to the RPC (*n* = 119) or control group (*n* = 99). Cows were maintained in a single pasture on Bahia grass and fed ~16 kg/cow/d (as fed) of a supplement comprised of: 60% corn silage, 30% cotton gin byproduct and 10% cottonseed meal (w/w) (all in as fed basis). Ovulation was synchronized with a 7-d Cosynch-CIDR protocol for TAI. Cows received 100 µg gonadotropin releasing hormone (GnRH, Factrel, Zoetis, Parsippany, NJ, USA) intramuscularly (i.m.) and an intravaginal CIDR device (1.38 g progesterone, Eazi-Breed CIDR Cattle Insert; Zoetis Inc., Madison, NJ, USA) insertion on d −9 of the protocol. On d −2, CIDR was removed and 25 mg prostaglandin F2α (Lutalyse, Zoetis) was injected, i.m. On d 0, 100 µg GnRH was administered i.m. and insemination was performed at the same time with conventional semen from one of a total of 6 sires. Cows were randomly assigned to sire within breed.

In addition to the diet listed above, each cow was given access to the assigned experimental diet of 514 g consisting of either 60 g RPC (equivalent to 17.3 g choline chloride) in 454 g ground corn gluten or 514 g ground corn gluten (control) through the Super SmartFeed machine. Experimental diets were fed individually for 9 d using a single Super SmartFeed machine. During the experimental feeding period, two of the bins had RPC supplement and the other two bins had control supplement. The feed was released to a cow if it approached either of the feeding bins for the assigned diet. The machine was programmed to allow each cow to consume no more than 514 g of the diet each day. The experimental diet was available from d −1 until d 7 relative to TAI. This represents a period corresponding to final follicular growth, ovulation, fertilization and embryonic development until the blastocyst stage of development. Each cow had the opportunity to consume up to 514 g of the assigned experimental diet each day; some animals voluntarily ate less.

Pregnancy diagnosis was performed by transrectal ultrasonography at d 28 and 42 after insemination. Blood samples (10 mL) were collected by jugular venipuncture at d 28, 42, 76, 150 and 220 (relative to TAI) into EDTA-containing tubes to measure the plasma concentration of pregnancy-associated glycoproteins (PAG) in pregnant cows. Blood samples were centrifuged after collection at 3,000 ×* g* for 15 min to obtain the plasma fraction. Plasma samples were stored at −80 °C until further analysis. All plasma samples were subjected to an in-house validated sandwich enzyme-linked immunosorbent assay for quantification of PAG [17]. The intra- and inter-assay coefficients of variation were 5.59% and 12.22%, respectively.

Pregnant cows were kept on Bahia grass pastures under similar managemental conditions until calving. Data on gestation length, calf sex, and birth weight were collected at calving. At an average age of 118 d (range 75–150 d), body weight and hip height data were collected from all calves. Bull calves were castrated on the same day and paired testes weight was recorded after removing all external connective tissue. All calves were weaned on the same day (average age of 248 d, range 205–280 d) and data were collected on body weight and hip height. Adjusted 205-d weaning weight was calculated using the formula [(weaning weight − birth weight)/days of age at weaning] × 205 + birth weight.

### Statistical analysis

Data were analyzed using the Statistical Analysis System version 9.4 (SAS Institute, Cary, NC, USA). Data on plasma choline metabolites in Exp. 1 were analyzed by analysis of variance using the MIXED procedure of SAS. The statistical model included the fixed effects of treatment, day, and day by treatment interaction, the random effect of cow nested within treatment and with two covariates (daily intake of the experimental diet and the interval between the last meal of the experimental diet before blood sampling and the time of blood sample collection).

For Exp. 2, cows that consumed less than 150 g/d of the experimental ration (*n* = 9) or that gave birth to twin calves (*n* = 1) were removed from the experiment. The daily feed intake of the remaining 208 cows as recorded by the Super SmartFeed machine averaged 216 g for control (quartiles 1, 2 and 3 = 206, 231, and 241 g), and 206 g for RPC (quartiles 1, 2 and 3 = 190, 223, and 237 g). The total number of calves born from the 208 cows was 89 (20 bull calves and 21 heifer calves from control; 22 bull calves and 26 heifer calves from RPC). Of the 89 calves, three (one bull calf from control and two heifer calves from RPC) died after birth. Only the data on birth weight was included in analyses for those three calves. Data for binomial variables (i.e., pregnancy outcomes) collected in Exp. 2 were analyzed by logistic regression using the GLIMMIX procedure of SAS with the logit link and binary distribution. The statistical model included the effects of treatment, sire, breed of dam, and replicate. Sire and breed were removed from the final model due to non-significant effects and *F*-value < 2. Continuous data (i.e., body weight, height, etc.) were analyzed by the PROC GLM procedure of SAS. Effects of sire and breed were not significant, and the final statistical model included the effects of treatment, replicate, treatment by replicate interaction, sex, sex by treatment interaction, dam age (3 years vs. > 3 years) and with age of calf as a covariate.

## Results

### Circulating concentrations of choline and its metabolites in cows fed in a self-feeding device (Exp. 1)

**Fig. 1:**
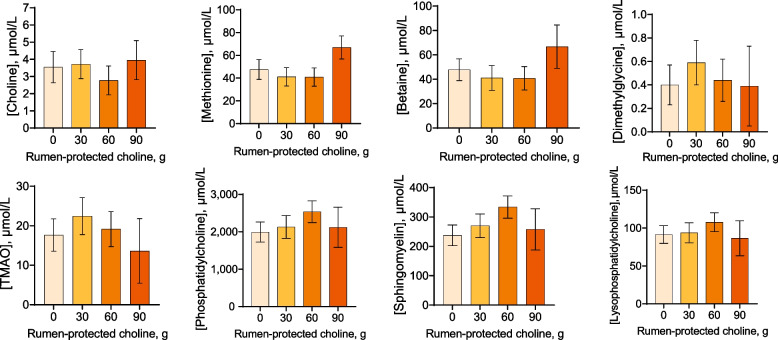
Effects of feeding rumen-protected choline (RPC) on plasma concentrations of choline and its metabolites (Exp. 1). A total of 24 postpartum suckled beef cows were randomly assigned to be individually fed either 0 (*n* = 5), 30 (*n* = 7), 60 (*n* = 7), or 90 (*n* = 5) g of RPC mixed in 454 g of ground corn gluten. Blood samples were collected at d −1, 5, 5.5, 10, and 10.5 relative to the start of feeding. Note that animals consumed the experimental diet free choice until an amount equal to 454 g plus the amount of choline had been consumed so the time of blood sample collection relative to time of last meal varied. Data are presented as least-squares means ± SEM. There were no differences between feeding groups or interactions between feeding group and day

The RPC feeding group did not affect concentrations of any metabolite (Fig. 1). There was also no interaction between treatment and the day of sample collection.

### Programming of postnatal development by feeding of rumen-protected choline in the periconceptional period (Exp. 2)

Treatment with RPC did not affect the proportion of cows becoming pregnant at d 28 or 42 post-AI or the proportion of cows calving (Table 1). Similarly, treatment did not affect pregnancy loss between d 28 and 42 of gestation, d 42 and calving, d 28 and calving (Table 1).

**Table 1:** Effects of feeding rumen-protected choline (RPC) during the periconceptional period on pregnancy outcomes and calving rate (Exp. 2)

Item	Control	RPC	95% confidence limits	*P-*value
Proportion of cows pregnant, % (*n/n*)				
Day 28	51.7 (47/91)	47.0 (55/117)	0.421–1.319	0.310
Day 42	49.4 (45/91)	43.6 (52/117)	0.426–1.324	0.321
Proportion of cows calved, % (*n/n*)	45.1 (41/91)	41.0 (48/117)	0.446–1.389	0.406
Pregnancy loss, % (*n/n*)				
Day 28–42	4.3 (2/47)	5.5 (3/55)	0.163–7.333	0.925
Day 42-calving	8.9 (4/45)	7.7 (4/52)	0.190–3.841	0.837
Day 28-calving	12.8 (6/47)	12.7 (7/55)	0.276–3.138	0.908

**Fig. 2:**
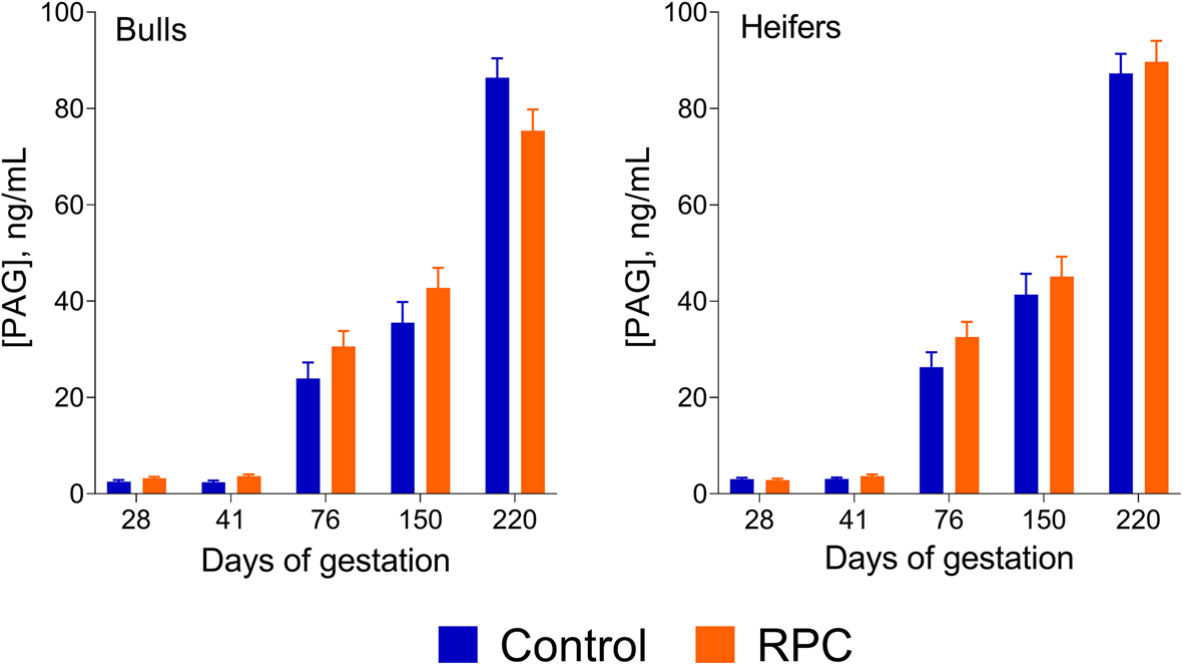
Effects of feeding rumen-protected choline (RPC) during the periconceptional period on plasma concentrations of pregnancy-associated glycoproteins (PAG) in Exp. 2. Blood samples were collected on d 28, 42, 76, 150, and 220 relative to timed artificial insemination. Data are presented as least-squares means ± SEM. There was no effect (*P* > 0.100) of RPC, sex, or the interaction on plasma concentrations of PAG on any of the days evaluated

The concentration of plasma PAG were measured at d 28, 41, 76, 150, and 220 post-AI. There was no effect (*P* > 0.10) of RPC, sex, or the interaction on plasma circulating levels of PAG (Fig. 2). There was also no effect of RPC (*P* = 0.760) on gestation length. There was a tendency for an interaction of RPC and calf sex (*P* = 0.077) with RPC reducing gestation length in males but increasing gestation length in females.

Data on postnatal phenotype of calves is presented in Table 2. Birth weight was not affected by treatment (*P* = 0.698), calf sex (*P* = 0.173) or the interaction (*P* = 0.640). At ~118 days of age, calves born from dams fed RPC were lighter in weight (*P* = 0.040) than control calves. There was no treatment by sex interaction (*P* = 0.119) although, numerically, the difference in weight was greater for bull calves (13 kg) than heifer calves (2 kg). Hip height at an average age of 118 d was not affected (*P* = 0.835) by treatment. There was a tendency for a treatment by sex interaction (*P* = 0.099). The weight-to-height ratio at 118 d was lower in RPC calves (*P* = 0.012) and there was no interaction with sex (*P* = 0.320). Paired testis weight from bull calves was not affected by feeding (*P* = 0.679).

**Table 2:** Effects of feeding rumen-protected choline (RPC) during the periconceptional period on gestational length and postnatal phenotype of calves (Exp. 2)^a^

Item	Treatment				*P*-value		
	Control male^e^	RPC male^f^	Control female^g^	RPC female^h^	Trt	Sex	Trt × Sex
Gestation length, d	280.9 ± 1.1	278.6 ± 1.1	277.8 ± 1.1	279.4 ± 1.0	0.760	0.308	0.077
Birth weight, kg	32.4 ± 1.2	31.4 ± 1.2	30.2 ± 1.2	30.3 ± 1.1	0.698	0.173	0.640
118 d^b^							
Body weight, kg	153.3 ± 3.6	140.6 ± 3.4	146.1 ± 3.6	144.2 ± 3.3	0.040	0.598	0.119
Hip height, cm	96.02 ± 1.1	94.44 ± 1.1	93.88 ± 1.1	95.92 ± 1.0	0.835	0.765	0.099
Weight/height ratio, kg/cm	1.59 ± 0.03	1.48 ± 0.03	1.54 ± 0.03	1.49 ± 0.03	0.012	0.555	0.320
Testis weight, g	49.3 ± 4.2	44.44 ± 3.8	-	-	0.679	-	-
248 d (weaning)^c^							
Body weight, kg	252.5 ± 5.5	231.7 ± 5.1	244.1 ± 5.4	236.3 ± 5.0	0.008	0.716	0.220
Hip height, cm	114.53 ± 1.1	112.46 ± 1.0	112.26 ± 1.1	110.88 ± 1.0	0.104	0.744	0.744
Weight/height ratio, kg/cm	2.19 ± 0.04	2.05 ± 0.04	2.16 ± 0.04	2.12 ± 0.04	0.024	0.631	0.194
Adjusted weaning weight, kg^d^	234.5 ± 4.9	218.7 ± 4.6	224.2 ± 4.8	219.9 ± 4.4	0.037	0.228	0.228

^a^Data are least-squares means ± SEM

^b^Data were collected on a specific date and at ages ranging from 75 to 150 d. Age at collection was considered as a covariate in the statistical analysis

^c^Data were collected on a specific date and at ages ranging from 205 to 280 d. Age at collection was considered as a covariate in the statistical analysis

^d^Adjusted 205-d weaning weight was calculated using the formula [(weaning weight − birth weight)/days of age at weaning] × 205 + birth weight

^e^*n* = 20 for gestation length and birth weight and *n* = 19 for other traits

^f^*n* = 22

^g^*n* = 21

^h^*n* = 26 for gestation length and birth weight and *n* = 24 for other traits

The effects of feeding RPC to dams on the body weight of calves persisted until weaning at an average age of 248 d. This was true when evaluating weight at weaning (*P* = 0.008) or 205-d adjusted weaning weight (*P* = 0.037). The treatment by sex interaction was not significant for weaning weight (*P* = 0.220) or adjusted weaning weight (*P* = 0.228) but the difference between feeding groups was numerically greater for bulls than heifers (21 vs. 8 kg for weaning weight and 16 vs. 4 kg for adjusted weaning weight). Hip height tended (*P* = 0.104) to be less for RPC calves than for control calves regardless of sex (interaction, *P* = 0.744). The weight-to-hip ratio was also lower (*P* = 0.024) for the RPC group. The treatment by sex interaction was not significant (*P* = 0.194).

## Discussion

The present study demonstrated that feeding RPC during the periconceptional period can program the preimplantation embryo to result in alterations in postnatal phenotype that include reduced body weight at 118 and 248 days of age and weight-to-hip ratio. The actions of feeding RPC are opposite to what has been observed when preimplantation bovine embryos were cultured with choline chloride [11–13]. In those experiments, calves produced from choline-treated embryos experienced increases in body weight, paired testes weight, cross-sectional area of the *longissimus thoracis* muscle, and carcass weight. We conclude that the actions of choline in the current experiment are mediated by different mechanisms than for the cultured bovine embryo. This conclusion is based on two features of the earlier and current experiments. For experiments with cultured embryos, control embryos were cultured in the absence of choline chloride. In the present study, however, choline is present in the reproductive tract of control cows because it has been detected in oviductal [18] and uterine fluid [19]. In addition, feeding of RPC did not cause a detectable increase in circulating choline concentrations. Examination of studies in which RPC was fed to cows would indicate that changes in circulating choline concentrations after feeding are not consistently observed [20–25]. Even if a small rise in circulating concentrations occurred that was not detected because of the sampling regimen, that rise would be unlikely to have a meaningful change in reproductive tract concentrations of choline, which averaged 566 μmol/L in the oviductal fluid [18]. It should be noted that, on average, cows ate less of the experimental diet than was available, so the amounts of RPC consumed were less than planned.

The most likely explanation for the programming actions of feeding RPC was that other metabolites of choline or alterations in rumen function associated with feeding RPC served as developmental programming cues in the present study. One metabolite of choline that is a candidate for function as a programming molecule is TMAO, which was increased after oral administration of choline [23]. TMAO functions as an osmolyte and regulator of protein folding [26, 27]. Increased concentrations of TMAO have been associated with a variety of pathologies in humans including cardiovascular disease, kidney disease, cancer, and metabolic syndrome [27]. Actions of TMAO, such as activation of pro-inflammatory pathways such as NF-kB signaling [28] could lead to direct or indirect effects on the developmental program of the embryo. It is also possible that changes in the local production of TMAO in the rumen could result in changes in the function or composition of the ruminal microbiome or functional changes.

One can postulate three mechanisms by which feeding RPC in the preimplantation period altered postnatal phenotype. The first is direct actions of one or more metabolites of RPC on the embryo to affect subsequent development of the fetus or placenta. Effects of RPC on the embryo might involve changes in the epigenome of the early embryo. Supplementation of choline in embryo culture medium increased DNA methylation in bovine blastocysts [29] and resulted in changes in the methylome of the calf [12, 13]. Furthermore, feeding RPC during the transition period increased global DNA methylation in the whole blood for male calves but not female calves [30]. The second possibility is that RPC feeding changed maternal physiology (such as alterations in pituitary, ovarian, oviductal or uterine function) that altered development of the embryo. It may also be that programming of placental function by RPC, either through direct actions on the embryo or indirect actions mediated by changes in maternal physiology, resulted in changes in placental function that either decreased capacity of the fetus for postnatal growth or that decreased secretion of mammogenic molecules so that milk supply of the calf after birth was decreased. We did not measure circulating concentrations of mammogenic hormones during pregnancy or milk yield of cows after calving. Thus, the questions of whether RPC feeding during the preimplantation period affects mammogenesis remains unknown. Performing feeding experiments with dairy cattle, where calves do not gain their nutrition from their dams after birth, would be an important experiment to determine any involvement of lactation in the programming of postnatal growth by RPC.

Effects of RPC on plasma concentrations of PAG, which are produced by trophoblast cells [31], were assessed. It has been proposed that circulating concentrations of PAG can be marker of embryonic/fetal viability and placental function [31]. Concentrations of PAG in blood were lower for cows subsequently experiencing fetal loss [31]. Furthermore, feeding a low protein diet during the first trimester increased PAG concentrations in heifers [32] while feed restriction during pregnancy in sheep increased circulating PAG concentrations [33]. That there was no change in plasma concentrations of PAG is suggestive of a lack of effect of RPC on placental function although other aspects of placental function may have been modified.

The consequences of developmental programming are often different for males and females [34] although mechanisms involved are unknown. For instance, differences in weaning weight between calves derived from embryos treated with choline in vitro vs. control embryos tended to be greater for male calves than female calves [11]. The same tendency was observed in the present study although the interaction was not significant. More work with larger number of animals is required to determine whether sexual dimorphism in response to feeding RPC is a real effect.

## Conclusions

In summary, feeding RPC during the periconceptional period can program the preimplantation embryo to have postnatal consequences including a reduction in body weight, hip height, and weight-to-hip ratio of the calves. These actions of feeding RPC are opposite to what has been observed with provision of choline chloride to cultured preimplantation embryos. Given the lack of a detectable increase in circulating choline concentrations after supplementation of RPC, it is likely that other metabolites of choline or alterations in rumen function associated with feeding RPC served as developmental programming cues.

## Data Availability

Not applicable.

## Abbreviations

GnRH: Gonadotropin releasing hormone
i.m.: Intramuscularly
PAG: Pregnancy-associated glycoproteins
RFID: Radio frequency identification
RPC: Rumen-protected choline
TAI: Timed artificial insemination
TMAO: Trimethylamine *N*-oxide

## References

[1] Goyal D, Limesand SW, Goyal R. Epigenetic responses and the developmental origins of health and disease. J Endocrinol. 2019;242:105–19. 10.1530/JOE-19-0009. 10.1530/JOE-19-0009 31091503

[2] Watkins AJ, Lucas ES, Wilkins A, Cagampang FR, Fleming TP. Maternal periconceptional and gestational low protein diet affects mouse offspring growth, cardiovascular and adipose phenotype at 1 year of age. PLoS ONE. 2011;6:28745. 10.1371/journal.pone.0028745. 10.1371/journal.pone.0028745 PMC3240629 22194901

[3] Williams CL, Teeling JL, Perry VH, Fleming TP. Mouse maternal systemic inflammation at the zygote stage causes blunted cytokine responsiveness in lipopolysaccharide-challenged adult offspring. BMC Biol. 2011;9:49. 10.1186/1741-7007-9-49. 21771319 10.1186/1741-7007-9-49 PMC3152940

[4] Kannampuzha-Francis J, Denicol AC, Loureiro B, Kaniyamattam K, Ortega MS, Hansen PJ. Exposure to colony stimulating factor 2 during preimplantation development increases postnatal growth in cattle. Mol Reprod Dev. 2015;82:892–7. 10.1002/mrd.22533. 26227079 10.1002/mrd.22533

[5] Amaral TF, Gonella-Diaza A, Heredia D, Melo GD, Estrada-Cortés E, Jensen LM, et al. Actions of DKK1 on the preimplantation bovine embryo to affect pregnancy establishment, placental function, and postnatal phenotype. Biol Reprod. 2022;10:945–55. 10.1093/biolre/ioac128. 10.1093/biolre/ioac128 35765194

[6] Hedtke V, Bakovic M. Choline transport for phospholipid synthesis: An emerging role of choline transporter-like protein 1. Exp Biol Med. 2019;244:655–62. 10.1177/1535370219830997. 10.1177/1535370219830997 PMC6552397 30776907

[7] Jiang X, West AA, Caudill MA. Maternal choline supplementation: a nutritional approach for improving offspring health? Trend Endocrinol Metab. 2014;25:263–73. 10.1016/j.tem.2014.02.001. 10.1016/j.tem.2014.02.001 24680198

[8] Shen J, Sun B, Yu C, Cao Y, Cai C, Yao J. Choline and methionine regulate lipid metabolism via the AMPK signaling pathway in hepatocytes exposed to high concentrations of nonesterified fatty acids. J Cell Biochem. 2020;121:3667–78. 10.1002/jcb.29494. 31680310 10.1002/jcb.29494

[9] Wang C, Liu ZY, Huang WG, Yang ZJ, Lan QY, Fang AP, et al. Choline suppresses hepatocellular carcinoma progression by attenuating AMPK/mTOR-mediated autophagy via choline transporter SLC5A7 activation. Hepatobiliary Surg Nutr. 2024;13:393–411. 10.21037/hbsn-22-476. 38911213 10.21037/hbsn-22-476 PMC11190510

[10] France TL, Ortega AF, Richards AT, Farricker MJ, Fontoura AB, McFadden JW. Abomasal infusion of deuterium-labeled choline confirms that choline is a methyl donor in gestating and lactating Holstein dairy cattle. J Nutr. 2024;S0022–3166(24):01183. 10.1016/j.tjnut.2024.11.014. 10.1016/j.tjnut.2024.11.014 39581267

[11] Haimon MLJ, Estrada-Cortés E, Amaral TF, Block J, Jeensuk S, Maia TS, et al. A low concentration of choline chloride alters the developmental program of the bovine preimplantation embryo. Reprod Fertil. 2024;5:e240058. 10.1530/RAF-24-0058. 39361491 10.1530/RAF-24-0058 PMC11558960

[12] Estrada-Cortés E, Ortiz W, Rabaglino MB, Block J, Rae O, Jannaman EA, et al. Choline acts during preimplantation development of the bovine embryo to program postnatal growth and alter muscle DNA methylation. FASEB J. 2021;35:e21926. 10.1096/fj.202100991r. 34533870 10.1096/fj.202100991R PMC12316091

[13] Haimon ML, Estrada-Cortés E, Amaral TF, Martin H, Jeensuk S, Block J, et al. Provision of choline chloride to the bovine preimplantation embryo alters postnatal body size and DNA methylation. Biol Reprod. 2024;111:567–79. 10.1093/biolre/ioae092. 38857381 10.1093/biolre/ioae092

[14] Pinotti L, Baldi A, Dell’Orto V. Comparative mammalian choline metabolism with emphasis on the high-yielding dairy cow. Nutr Res Rev. 2002;15:315–32. 10.1079/nrr200247. 19087410 10.1079/NRR200247

[15] Holm PI, Ueland PM, Kvalheim G, Lien EA. Determination of choline, betaine, and dimethylglycine in plasma by a high-throughput method based on normal-phase chromatography–tandem mass spectrometry. Clin Chem. 2003;49:286–94. 10.1373/49.2.286. 12560353 10.1373/49.2.286

[16] Koc H, Mar MH, Ranasinghe A, Swenberg JA, Zeisel SH. Quantitation of choline and its metabolites in tissues and foods by liquid chromatography/electrospray ionization-isotope dilution mass spectrometry. Anal Chem. 2002;74:4734–40. 10.1021/ac025624x. 12349977 10.1021/ac025624x

[17] Pohler KG, Peres RF, Green JA, Graff H, Martins T, Vasconcelos JL, et al. Use of bovine pregnancy-associated glycoproteins to predict late embryonic mortality in postpartum Nelore beef cows. Theriogenology. 2016;85:1652–9. 10.1016/j.theriogenology.2016.01.026. 26928645 10.1016/j.theriogenology.2016.01.026

[18] Mahé C, Gatien J, Desnoes O, Le Bourhis D, Mermillod P, Salvetti P, et al. Metabolomic analysis of oviduct fluid on day 3 post-estrus in Holstein heifers. Reprod Biol. 2021;21:100512. 10.1016/j.repbio.2021.100512. 33991764 10.1016/j.repbio.2021.100512

[19] Tríbulo P, Balzano-Nogueira L, Conesa A, Siqueira LG, Hansen PJ. Changes in the uterine metabolome of the cow during the first 7 days after estrus. Mol Reprod Dev. 2019;86:75–87. 10.1002/mrd.23082. 30383328 10.1002/mrd.23082 PMC6322963

[20] Zenobi MG, Scheffler TL, Zuniga JE, Poindexter MB, Campagna SR, Gonzalez HC, et al. Feeding increasing amounts of ruminally protected choline decreased fatty liver in nonlactating, pregnant Holstein cows in negative energy status. J Dairy Sci. 2018;101:5902–23. 10.3168/jds.2017-13973. 29680650 10.3168/jds.2017-13973

[21] De Veth MJ, Artegoitia VM, Campagna SR, Lapierre H, Harte F, Girard CL. Choline absorption and evaluation of bioavailability markers when supplementing choline to lactating dairy cows. J Dairy Sci. 2016;99:9732–44. 10.3168/jds.2016-11382. 27771079 10.3168/jds.2016-11382

[22] Potts SB, Scholte CM, Moyes KM, Erdman RA. Production responses to rumen-protected choline and methionine supplemented during the periparturient period differ for primi-and multiparous cows. J Dairy Sci. 2020;103:6070–86. 10.3168/jds.2019-17591. 32359982 10.3168/jds.2019-17591

[23] France TL, Myers WA, Javaid A, Frost IR, McFadden JW. Changes in plasma and milk choline metabolite concentrations in response to the provision of various rumen-protected choline prototypes in lactating dairy cows. J Dairy Sci. 2022;105:9509–22. 10.3168/jds.2021-21615. 36241441 10.3168/jds.2021-21615

[24] Lima FS, Sá Filho MF, Greco LF, Santos JEP. Rumen-protected choline improves metabolism and lactation performance in dairy cows. Animals (Basel). 2024;14:1016. 10.3390/ani14071016. 38612255 10.3390/ani14071016 PMC11010861

[25] Arshad U, Zimpel R, Husnain A, Poindexter MB, Santos JEP. Effect of rumen-protected choline on fat digestibility and lymph metabolome in dairy cows. J Anim Physiol Anim Nutr (Berl). 2024;108(4):950–64. 10.1111/jpn.13943. 38379267 10.1111/jpn.13943

[26] Mukherjee M, Mondal J. Unifying the contrasting mechanisms of protein-stabilizing osmolytes. J Phys Chem B. 2020;124:6565–74. 10.1021/acs.jpcb.0c04757. 32633959 10.1021/acs.jpcb.0c04757

[27] Ilyas A, Wijayasinghe YS, Khan I, El Samaloty NM, Adnan M, Dar TA, et al. Implications of trimethylamine N-oxide (TMAO) and betaine in human health: beyond being osmoprotective compounds. Front Mol Biosci. 2022;9:964624. 10.3389/fmolb.2022.964624. 36310589 10.3389/fmolb.2022.964624 PMC9601739

[28] Seldin X, Meng Y, Qi H, Zhu W, Wang Z, Hazen SL, et al. Trimethylamine N-oxide promotes vascular inflammation through signaling of mitogen-activated protein kinase and nuclear factor-ĸB. J Am Heart Assoc. 2016;5:e002767. 10.1161/jaha.115.002767. 26903003 10.1161/JAHA.115.002767 PMC4802459

[29] Estrada-Cortés E, Negrón-Peréz VM, Tríbulo P, Zenobi MG, Staples CR, Hansen PJ. Effects of choline on the phenotype of the cultured bovine preimplantation embryo. J Dairy Sci. 2020;103:10784–96. 10.3168/jds. 32896407 10.3168/jds.2020-18598

[30] Holdorf HT, Brown WE, Combs GJ, Henisz SJ, Kendall SJ, Caputo MJ, et al. Increasing the prepartum dose of rumen-protected choline: Effects of maternal choline supplementation on growth, feed efficiency, and metabolism in Holstein and Holstein× Angus calves. J Dairy Sci. 2023;106:6005–27. 10.3168/jds.2022-23068. 37500446 10.3168/jds.2022-23068

[31] Wallace RM, Pohler KG, Smith MF, Green JA. Placental PAGs: gene origins, expression patterns, and use as markers of pregnancy. Reproduction. 2015;149:R115–26. 10.1530/REP-14-0485. 25661256 10.1530/REP-14-0485

[32] Sullivan TM, Micke GC, Magalhaes RS, Martin GB, Wallace CR, Green JA, et al. Dietary protein during gestation affects circulating indicators of placental function and fetal development in heifers. Placenta. 2009;30:348–54. 10.1016/j.placenta.2009.01.008. 19233467 10.1016/j.placenta.2009.01.008

[33] Barbato O, Barile VL, Menchetti L, Ricci G, Achihaei EL, Porcu C, et al. Maternal undernutrition effect on pregnancy-associated glycoprotein (PAG) concentration in sheep carrying single and multiple fetuses. Animals (Basel). 2024;14:3427. 10.3390/ani14233427. 39682391 10.3390/ani14233427 PMC11639935

[34] Hansen PJ, Dobbs KB, Denicol AC, Siqueira LG. Sex and the preimplantation embryo: implications of sexual dimorphism in the preimplantation period for maternal programming of embryonic development. Cell Tiss Res. 2016;363:237–47. 10.1007/s00441-015-2287-4. 10.1007/s00441-015-2287-4 PMC4703572 26391275

